# The Prevalence and Management of Chronic Tonsillitis: Experience From Secondary Care Hospitals in Rabak City, Sudan

**DOI:** 10.7759/cureus.34914

**Published:** 2023-02-13

**Authors:** Mujtaba Alrayah

**Affiliations:** 1 Otolaryngology - Head and Neck Surgery, Al-Baha University, Al-Baha, SAU; 2 Otolaryngology, Faculty of Medicine, El Emam El Mahadi University, Al-Baha, SAU

**Keywords:** tonsillectomy, sore throat, rabak city, incidence, chronic tonsillitis

## Abstract

Background

Tonsillitis is defined as an inflammation of the tonsils, which is a common clinical condition caused by either bacterial or viral infections. It affects a significant percentage of the population especially children. Chronic tonsillitis (CT) is described as when an individual suffers from seven or more attacks of tonsillitis per year.

Aim

This study aimed to determine the prevalence and management of CT among patients attending all secondary care hospitals in Rabak city, Sudan.

Methods

A cross-sectional descriptive study was conducted in June-September 2022. A structured questionnaire was used to screen 297 Patients who presented to all ENT clinics within the study period. Out of the 297 patients, 77 patients were confirmed to be having CT based on the inclusion criteria. Data collected were analyzed using SPSS version 21 and arranged into a simple frequency table.

Results

The prevalence of CT was found to be 25.9 % (77) among all screened patients. The majority (67.5%) of the patients with CT were male, and mostly between the age of 11-20 years (32.4%). A considerable number of them (32.4%) were residing in the urban-industrial part of the city, and about 36.3% are not working. All the patients with CT complained of throat pain, while 88% presented with red inflamed tonsils. Laboratory investigations of these patients revealed 64% to have Neutrophilia, while 54 and 50% had high ESR and positive ASO titer, respectively. 57% of the patients underwent tonsillectomy while (30%) were managed medically.

Conclusion

The prevalence of CT was found to be high in the agro-industrial area of Rabak city and more among teenagers, and most cases were managed by tonsillectomy.

## Introduction

Ear, nose, and throat (ENT) disease particularly tonsillitis represent a considerable health burden in Sudan with the high cost to the Sudanese National Health Service. This is due to high incidence, cost of treatment, and complications [1]. Understanding the knowledge and magnitude of ENT diseases will aid the health authorities to implement its management and preventive programs.

Tonsillitis is inflammation of the tonsils, a common clinical condition caused by either bacterial or viral infection [2,3]. It affects a significant percentage of the population, especially children. Chronic tonsillitis (CT) is described as when an individual suffers from seven or more attacks of acute tonsillitis per year [4]. Acute tonsillitis is characterized by visible streaks of pus or cheesy material on the tonsillar surface, and the entire tonsil may become enlarged and hyperemic suggestive of an inflammatory process. Tonsillitis is caused mainly by β-hemolytic Streptococcus, called strep throat, and to a lesser extent by Staphylococcus aureus and several other bacteria. The more common symptoms of acute tonsillitis are sore throat, red swollen tonsils, pain when swallowing, fever, cough, headache, tiredness, chills, swollen lymph nodes in the neck, and pain in the ears or neck, and the less common symptoms include nausea, stomachache, vomiting, furry tongue, bad breath, and change in voice and difficulty in opening the mouth [5]. There are three approaches to the management of CT: conservative, use of antibiotics, or by tonsillectomy. Surgical removal of the tonsils provides the definitive treatment [6].

Although the CT rate was expected to be high in the agro-industrial area of Rabak city in Sudan because of the environmental pollution, there are not enough data on its epidemiology in the area. This study aimed to determine the prevalence, clinical features, laboratory findings, and treatment modalities of patients with CT among patients reporting to secondary care hospitals in Rabak city, Sudan.

## Materials and methods

Ethical considerations

This research was approved by the Ministry of Health Research Committee, White Nile province, with ethical clearance no (5). Moreover, verbal notified consent was obtained from all participants and or their informants.

Study design

A cross-sectional, observational hospital-based research was performed in the time of June to September 2022 including all patients who visited the secondary ENT hospital of Rabak city, Sudan, who suffered from sore throats (297). Patients were seen by two ENT consultants, and diagnosis of CT was done in the ENT clinics using a head mirror, light source, and tongue depressors. Any patient with a sore throat and who had seven or more attacks of acute tonsillitis was diagnosed with CT (77 out of 297). Demographic data, clinical features, lab investigations, and treatment modalities of CT were reported in this study.

Study population

Patients visiting Rabak secondary ENT hospital complaining of sore throat and who were diagnosed later with chronic tonsillitis (seven or more attacks of acute tonsillitis) during the research period who fit the research inclusion criteria were joined voluntarily.

Inclusion Criteria

The research inclusion criteria comprise both sex and all age groups, who lived in Rabak town, Sudan, and who were diagnosed with CT by ENT consultants. The whole number of permitted participants was 77 out of 297 patients.

Exclusion Criteria

The study exclusion criteria comprise any participants with deficient investigations. (n=3) were excluded.

Sample size estimation

The formula of sample size calculation (N = PQZ2/d2) was used to calculate the sample size (N = 77).

Where N = sample size, P = prevalence of CT disorders factor, Q = 1−p, Z = constant 95% occurred 1.96, and d = desired margin.

Data collection procedure

Data were collected using a structured questionnaire. The participants were approached with questions concerning their sociodemographic details, and ENT-related complaints such as sore throat (estimated with a scale system of severity graded from 1 to 10), whilst the ENT professionals applied and reported clinical examinations, ENT-related diagnosis, investigations, and management. Moreover, checking the records of the treated patients. All patients had gone through a comprehensive history and a full physical examination by ENT professionals. Relevant investigations were carried out according to the patient's complaint.

Data analysis

The patients’ data were processed statistically using the SPSS version 21. A descriptive statistical summarization was performed. The result was considered significant at a p-value less than 0.05.

## Results

A total number of 297 patients were screened within the study period out of which 77 (25.9 %) patients were diagnosed with CT. The majority 22 (28.6%) of patients were between the age of 11-20 years (Table 1).

**Table 1:** Age characteristics of study participants with CT in Rabak city, Sudan (n=77).

Age	Frequency/Percentage
< 10	6(7.8)
11 -20	22(28.6)
21-30	20(26)
31-40	15(19.5)
41-50	12(28.6)
>50	2(2.6)

Males accounted for 52 (67.5%) while females accounted for 25 (32.4%) of the patients (Table 2).

**Table 2:** Sex characteristics of study participants with CT in Rabak city, Sudan (n=77).

Sex	Frequency/Percentage
Male	52(67.5)
Female	25(32.4)

Most of the patients 25 (32.4%) lived in the urban-industrial part of the city (Table 3).

**Table 3:** Residence characteristics of study participants with CT in Rabak city, Sudan (n=77).

Residence	Frequency/Percentage
Urban	20(25.9)
Urban-industrial	25(32.4)
Rural	16(20.9)
Rural industrial	12(15.5)
Others	5(6.4)

While most of the patients 28 (36.3%) had no work to do, a very appreciable percentage 26(33.7%) were found to be working (Table 4).

**Table 4:** Occupation characteristics of participants with CT in Rabak city, Sudan (n=77).

Occupation	Frequency/Percentage
Industrial worker	2(2.6)
Farmer	5(6.5)
Office job	4(5.2)
Marginal employment	7(9)
Security office	1(1.3)
Others	4(5.2)
Not working	28(36.3)
Students	26(33.7)

All patients with CT 77 (100%) presented with complaints of throat pain, and during examination of the oropharynx, 88% of them presented with red swollen tonsils (Table 5).

**Table 5:** CT symptoms percentage among the participants with CT in Rabak city, Sudan (n=77).

CT symptoms	Percentage
Sore throat	100
Fever	67
Odynophagia	46
Constitutional symptoms	56
Red swollen tonsils	88
Jugulodigastric lymph nodes	65

Regarding lab investigations, 64% of patients showed neutrophilia in their blood film, while 54 and 50% had high ESR and positive ASO titer, respectively (Table 6).

**Table 6:** CT lab investigations percentage among the participants with CT in Rabak city, Sudan (n=77).

Lab. investigations	Percentage
Neutrophilia (+ve/-ve)	64
ESR (+ve/-ve)	54
ASO Titer	50
Low HB	22

Most of the patients (44%) underwent tonsillectomy while 30% were managed medically (Table 7).

**Table 7:** CT treatment modality percentage among participants with CT in Rabak city, Sudan (n=77).

Treatment modality/Frequency/Percentage	
Medical	30(39%)
Surgical	44(57%)
Referred	3(4%)

## Discussion

The incidence rate of CT was expected to be high among Sudanese in the area of Rabak city. This might be due to environmental pollution as in this area there are many factories and farms. Its exact rate, clinical features, and management are not well-reported.

The total number of participants who were diagnosed with CT was 77 which accounted for 25.9% of all patients attending ENT clinics at secondary care facilities in Rabak city, Sudan. This is higher than all ENT diseases attending our clinics in Rabak city. More so, the prevalence we got is higher than what was obtained by Anekpo and Modebe in a retrospective study done in Nigeria where the incidence rate was 2.7% out of 396 patients [7]. Our incidence rate was equally found to be higher than what Nanda and Bhalke obtained in a retrospective study done in India involving 690 patients where the rate was found to be 15.5% [8].

The present study revealed that the majority of the participants suffering from CT were teenagers between the age of 11-20 years. This is in line with a cross-sectional hospital-based study done in India by Sarode D and Bhole A who concluded that CT is more prevalent in the age group of 11-20 years [9]. The present findings are contrary to those determined by Mattila et al. who reported CT is more prevalent among adults above 20 years of age, while lesser in those less than 10 years of age [10]. In this study this may likely be due to missed diagnosis as most patients less than 10 years are seen by pediatricians not by ENT professionals, as such, they may miss the diagnosis. In relation to a previous study, the age group of 10-20 years is also reported to have a high incidence of CT, and this finding was explained by their low immunity, and cross-infection because of overcrowded classrooms and poor ventilation of the classrooms [3].

In this study, CT was found to be higher among the male gender residing in the urban-industrial part of Rabak city, this finding is in contrast to the study done by Abouzied and Massoud [11] as well as Bismi et al. [12] who reported female preponderance in their studies. Males are frequently stayed outdoors and exposed to infecting microorganisms and have more direct contact with diseased people, as well as the possibility of having allergies attributed to exposure to urban-industrial pollution in Rabak city. All these factors could likely be the reason for the male preponderance in this study.

A considerable number of the patients 28 (36.3%) with CT in this study are unemployed, and this could actually translate to poor nourishment, unhygienic condition, illiteracy, and improper medical care. The present findings are in line with Somro et al., who stated that CT is more prevalent among populations with low socioeconomic status [3].

The current study revealed that sore throat was a presenting symptom among all patients, this is in line with a prospective study undertaken by Batra et al. among 50 patients with CT [13]. Regarding laboratory investigations that were carried out among the studied patients, 64% showed neutrophilia in their blood film, while 54% and 50% have high ESR and positive ASO titer, respectively. All these findings were relatively high which are all in consonance with a descriptive study done by Roos, who reported that total WBC count, polymorphonuclear leucocytes, ESR, and ASO titer were found to be high in the majority of their study population with CT [14]. The marked neutrophilia and high ESR among the majority of the patients in this study may be due to the fact that infective causes are the leading cause of tonsillitis among our patients. Moreso, ASO titer was high among these patients suffering from tonsillitis, this may be a predictor of the known complications of Group A beta-hemolytic streptococci (GABS), which are rheumatic heart disease and glomerulonephritis.

Pal'chun and on the other hand Alasmari et al. stated that patients with fewer attacks of CT from GABA infection are best treated conservatively with penicillin and co-amoxiclav if there are no complications [15,16]. The surgical modality of treatment is the mainstay of treatment for patients with frequent attacks of CT as this proved to be the suitable modality for avoiding complications and averting future costs of medical treatment [17,18]. These previous conclusions explain the diversity of the treatment modalities in the current study.

Limitations

This study has some potential limitations. First, the small size of the study patients. Second, the patients recruited are mostly men with few women involved. Finally, this study did not implement methodologies for bacterial investigation using cultures or rapid diagnostic tests to rule out GABS.

## Conclusions

In this study, the majority of the patients with CT are teenagers, residing within urban-industrial areas, and are unemployed. Most of them having CT are males. The majority presented with a sore throat and Red Swollen Tonsils on throat examination. Most patients show high Neutrophilia, high ESR, and ASO titer on laboratory investigations. CT treatment modalities vary from medical treatment to surgical tonsillectomy.

Recommendation

We recommend that Governmental projects and educational programs are crucial for improving the health system in Rabak city, and these should be implemented to hasten solutions to some health challenges like CT. Furthermore, prevention of tonsillitis particularly among pediatric age groups with overcrowded poor living conditions should be encouraged to reduce the risk and complications of CT. Finally, there is a need for further research in Rabak city to determine the risk factors for the high incidence of CT in Rabak city.
